# Effect of prior follicular wave synchronization and eCG on ovum pick-up and *in vitro* embryo production in Braford cows

**DOI:** 10.1590/1984-3143-AR2025-0020

**Published:** 2025-11-14

**Authors:** Felipe Gustavo Garcia, Bento Martins de Menezes, Caroline Fernandes Possebon, Rosana Klaus, Marcelo Silveira Albornoz, Janislene Mach Trentin, Daniele Missio, Daniela dos Santos Brum, Fabio Gallas Leivas

**Affiliations:** 1 Laboratório de Biotecnologia da Reprodução – BIOTECH, Universidade Federal do Pampa – UNIPAMPA, Uruguaiana, RS, Brasil; 2 Laboratório de Fisiologia Molecular e Integrativa da Reprodução – MINT, Universidade Federal do Pampa – UNIPAMPA, Uruguaiana, RS, Brasil; 3 In Vitro Rio Grande do Sul, Santana do Livramento, RS, Brasil; 4 Laboratório de Reprodução Animal, Universidade Federal do Paraná – UFPR, Palotina, PR, Brasil; 5 Programa de Redes Inovadoras de Tecnologias Estratégicas do Rio Grande do Sul ­– RITEs-RS, Palmeira das Missões, RS, Brasil

**Keywords:** IVP, Bos taurus taurus, superovulation, OPU, blastocysts

## Abstract

The aim of this study was to evaluate the effect of follicular wave synchronization and equine chorionic gonadotropin (eCG) prior to ovum pick-up (OPU) in Braford cows on the oocyte competence, maturation rate, and *in vitro* embryo production. Cows (n = 27) were divided into three groups on a crossover model: no treatment prior to OPU (Control), follicular wave synchronization (Synchro), and synchronization plus 800IU of eCG (eCG800). Donors of the groups Synchro and eCG800 were synchronized with 2 mg of estradiol benzoate (EB), prostaglandin F2α analogue (PGF2α) and intravaginal device with 1g of progesterone (P4) on D0. On day 3, eCG800 group donors received 800IU of eCG. On day 6, OPU was performed, and the number of follicles were counted and classified by diameter in small, medium, and large. In experiment 1, the viable oocytes were evaluated for competence development, nuclear maturation, and mitochondrial reorganization. In experiment 2, oocytes were matured, fertilized, and cultured *in vitro* to blastocyst stage. All analysis was performed by ANOVA, and the differences were compared by Tukey's test with significance *P* ≤ 0.05. The use of 800 IU of eCG increased (P < 0.05) the number of medium and large follicles compared to the Syncro group. The oocyte recovery, viability, nuclear or cytoplasmic maturation, cleavage, and grade 1 embryos rate did not differ among groups (P > 0.05). The blastocyst rate on D7 showed tendency (P = 0.075) to improve from Control (17±6.08%) to Synchro (23.8±8.95%) to eCG800 (37.3±6.51%). The dose of 800 IU of eCG 72 h before OPU increased the proportion and number of medium and large follicles in relation to the Control and Synchro groups, without affecting oocyte competence and tending to produce more blastocysts on D7.

## Introduction

A critical factor that influences *in vitro* embryo production (IVP) efficiency is the estrous cycle’s phase that ovum pick-up (OPU) is performed (Bó et al., 2019). Most of the commercial programs, the OPU is realized on a random day of the estrous cycle, with no control of the emergence of the follicular wave (Pontes et al., 2011; Morotti et al., 2014) and all visible follicles on ultrasound are aspirated.

A study presented satisfactory results when OPU was performed three to four days after estrous (Pieterse et al., 1991). Therefore, follicular wave synchronization prior to OPU improves oocyte competence (Cavalieri et al., 2018; Seneda et al., 2020; Nawaz et al., 2022). In this sense, the use of hormonal therapies such as equine chorionic gonadotropin (eCG) with a safe dose of 800 IU stimulates follicular growth, reduces polyspermy and improves cleavage rates (Ribas et al., 2018). This occurs because there is better oocyte quality due to the absence of atretic events (Bacelar et al., 2010). Follicular aspiration on random days of the estrous cycle, up to 85% of oocytes present some degree of atresia (Hendriksen et al., 2000).

*In vitro* embryo production is widely used, but blastocyst rate reached a plateau of 30 to 40%, very similar to what was found in the 1990s (Seneda et al., 2020). One of the factors that may influence these results is the intrinsic reproductive potential of the donor female, for which the antral follicle population is an important parameter. The antral follicle population predicts the superovulatory response of an animal, i.e., females with a larger antral follicle population produce more embryos in response to a superovulation protocol (Center et al., 2018). In taurine cows, the antral follicle count is lower compared to zebu cows (Sartori and Barros, 2011) resulting in less potential for production and viable embryos (Singh et al., 2004; Ireland et al., 2007; Silva-Santos et al., 2014).

Knowledge of the response of taurine cows to stimulation and the effect of eCG treatment on oocyte maturation and embryonic development needs to be further elucidated to improve IVP results. In this study, we hypothesized that synchronization of the follicular wave and ovarian follicular stimulation protocol using 800 IU eCG prior to OPU improves competent oocyte recovery and IVP from Braford donors (3/8 *Bos taurus indicus* and 5/8 *Bos taurus taurus*). Therefore, the aim of this study was to evaluate the effect of follicular wave synchronization and 800 IU of eCG prior to OPU in Braford cows on quantity and quality of oocytes for IVP.

## Methods

Chemicals and reagents were purchased from Sigma Chemical Co. (St. Louis, MO, USA) unless otherwise indicated; synchronization protocol hormones were obtained from Zoetis (São Paulo, SP, Brazil).

### Experimental location and animals

The experiment was approved and performed according to the National Council of Control in Animal Experimentation of Brazil (CONCEA, CEUA Unipampa), registered under protocol number 033/2021. The experiments were performed in two commercial farms, in Uruguaiana (experiment 1) and Santana do Livramento (experiment 2), Rio Grande do Sul, Brazil. The first experiment was between August and September of 2021 (winter in the Southern Hemisphere) and the second experiment was between April and May of 2022 (autumn in the Southern Hemisphere). Twenty-seven Braford cows (3/8 *Bos taurus indicus:* Brahman and 5/8 *Bos taurus taurus*: Hereford) aged 4 years were used. The cows had a body condition score of 5 (1 to 9 scale; Spitzer, 1986). Cows were fed grazing regimen, water and mineral supplementation *ad libitum*, under the same feeding and management conditions.

#### Experiment 1 – evaluation of oocyte competence and *in vitro* maturation (IVM)

**Figure 1:**
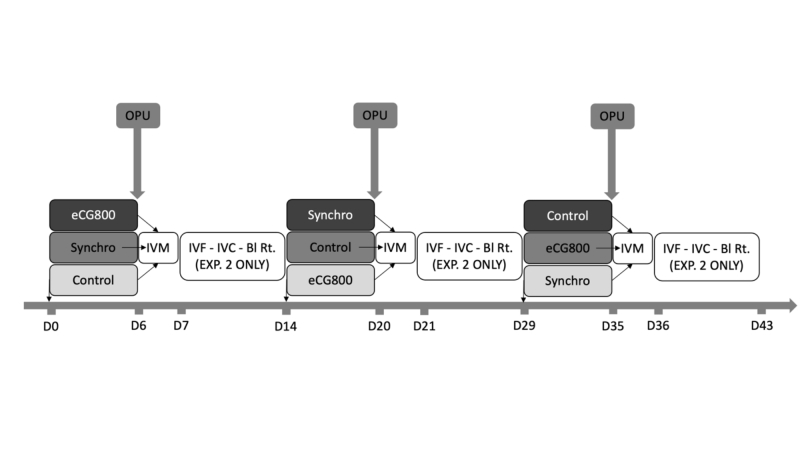
Experimental design used to evaluate the effect of follicular wave synchronization and eCG administration in Braford cows.OPU: Ovum pick-up; IVM: In Vitro Maturation (for both experiments); IVF: In Vitro Fertilization; IVC: In Vitro Culture; Bl Rt.: Blastocyst Rate (for experiment 2 only); Treatments: Control - cows submitted to OPU on random day of estrus cycle; Synchro - cows synchronized follicular wave prior OPU; eCG800 - cows synchronized follicular wave plus 800 IU of eCG prior OPU.

Fifteen primiparous Braford cows were randomly allocated into three groups according to treatments: Control: aspirated at random day of estrus cycle (n = 5); Synchro: with a pre-synchronized follicular wave (n = 5), and eCG800: pre-synchronized follicular wave plus 800IU of eCG three days before OPU (n = 5). A crossover model was used to reduce the influence of individual variation. Thus, all donors underwent the three treatments and follicular aspirations were performed at 14-day intervals (three replicates). The number of follicles were counted and classified by diameter, and the viable oocytes were evaluated for competence development, nuclear maturation and mitochondrial reorganization after 24 h of IVM (Figure 1).

#### Experiment 2 – evaluation of blastocyst rate

Twelve multiparous Braford cows were randomly allocated into three groups identical to experiment 1 (Control, Synchro and eCG800). On the day of the OPU, the number of follicles were counted and classified by diameter. Oocytes aspirated were matured, fertilized and embryos cultured *in vitro* for seven days to evaluate the blastocyst rate (Figure 1).

### Donor hormonal protocol

**Figure 2:**
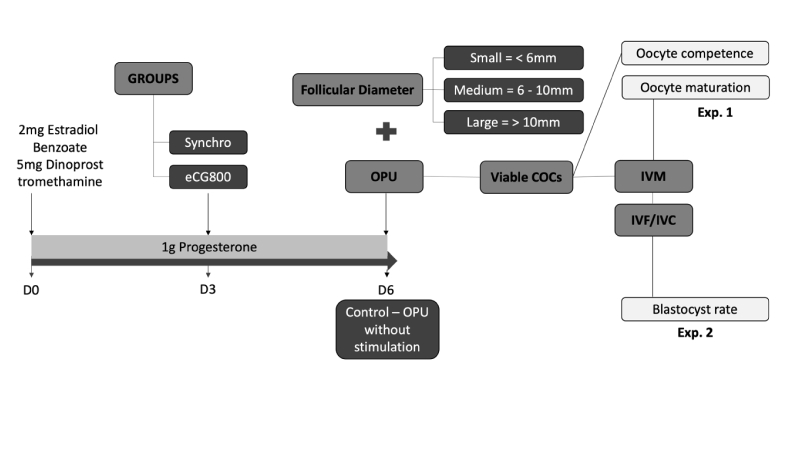
Hormonal protocol applied to Braford cow donors. Day 0: Control group did not receive any synchronization nor stimulation. Treatment groups received an intravaginal device with 1 g of progesterone, 2 mg of estradiol benzoate IM, and 25 mg of dinoprost tromethamine IM. Day 3: Separation of donors into one of the treatment groups: eCG800 (800 IU of eCG IM) or Synchro (no eCG treatment); Day 6: Removal of the intravaginal device, evaluation and of follicular diameter, follicular aspiration, oocyte selection, evaluation of oocyte competence development, beginning of IVM. In experiment 2, oocytes were fertilized and cultured, with cleavage and blastocyst rates evaluated.

Braford cows received on day 0 (D0) intramuscular (IM) 2 mg of estradiol benzoate (EB; Gonadiol®, Zoetis, Brazil), 12.5 mg of dinoprost tromethamine IM (Lutalyse®, Zoetis, Brazil) and an intravaginal device with 1g of progesterone (P4; CIDR®, Zoetis, Brazil). On day 3 (D3), donors of eCG800 received 800 IU of eCG. On day 6 (D6), the intravaginal P4 device was removed from groups Synchro and eCG800, and donors were subjected to OPU, through ultrasonic-guided follicular aspiration. The Control group did not receive any synchronization or stimulation (Figure 2).

### Follicular diameter, OPU, and oocyte classification

Prior to follicle aspiration, each cow received caudal epidural anesthesia using 4 mL of 2% lidocaine (Lidovet®, Bravet, Brazil). Feces were removed from the rectum and the perineum was cleaned with water and 70% ethanol. Immediately, before OPU, each ovary was located by rectal palpation and examined by ultrasonography using a portable ultrasound machine (Mindray Z5, Shenzhen, China) with a 5 MHz convex transducer attached in a plastic/acrylic vaginal probe guide (WTA, Cravinhos, SP, Brazil). All visible follicles were measured and classified according to the diameters (small: < 6 mm, medium: 6 to 10 mm, and large: > 10 mm, Machatkova et al., 2004). For OPU, a 20G aspiration needle (WTA, Cravinhos, SP, Brazil) was used which was connected to a conical tube (50 ml, Falcon, USA) via 1.20m silicone tubing (WTA, Cravinhos, SP, Brazil). Aspiration was performed at a vacuum pressure of 110 mmHg, equivalent to a flow rate of 10 to 15 mL of H_2_O/min, generated by a vacuum pump (WTA, Cravinhos, SP, Brazil). Dulbecco's Modified Phosphate-Buffered Saline (DMPBS) aspiration medium (Nutricell, Campinas, SP, Brazil) was supplemented with heparin (5 IU/ml). The collection conical tube with the medium was placed in a tube heater (WTA, Cravinhos, SP, Brazil) at a temperature of 37.5°C during the procedure. Each tube was identified with the donor number in addition to the date and time of aspiration. Donors were aspirated on the same day by the same operator.

### Oocyte selection

In the laboratory, after each aspiration the recovered follicular fluid was filtered (filter with 100 micron nylon screen, WTA, Cravinhos, SP, Brazil), and subsequently washed in the same medium in that they were collected. *Cumulus-oophorus* complexes (COCs) were selected under a stereomicroscope and were classified on the characteristics of the cumulus and ooplasm cells (Lonergan, 1992). Grade 1, 2 and 3 oocytes were selected for evaluation of competence and IVM. The number of COCs collected per OPU and the number of viable COCs recovered per OPU were quantified for each treatment.

### Evaluation of oocyte competence

In experiment 1, immediately after collection, the selected oocytes were washed three times in drops of DMPBS with the addition of 0.4% bovine serum albumin and pyruvate (2µL/ml). Then, the COCs were exposed to 26 µM of brilliant cresyl blue (BCB) diluted in DMPBS and incubated in an oocyte transporter (TO-16i, WTA, Cravinhos, SP, Brazil), in 1.5 mL microtubes (Eppendorf, Hamburg, Germany) for 90 minutes at 37.5ºC. Oocytes that remained blue were considered competent while colorless ones were not (Pujol et al., 2004).

### *In vitro* maturation

In experiment 1, oocytes were transported in a carrier (TO-16i, WTA, Cravinhos, SP, Brazil), organized in groups of COCs of the same treatment in 1.5 mL microtubes (Eppendorf, Hamburg, Germany) in TCM199 HEPES medium. In the laboratory, well plates (Nunc, Massachusetts, USA) matured with TCM199 plus 10% estrous mare serum, 5 µg/ml of follicle-stimulating hormone (FSH), 50 µg/ml of luteinizing hormone (LH), 22 µg/mL pyruvate and gentamicin. The maturation period was 21 to 24 h in an incubator at 38.7ºC, in a gaseous atmosphere with 5% CO_2_ and saturated humidity. On experiment 2, oocytes of each donor were put into 5 mL glass test tubes with 300 µL of maturation media (ABS Pecplan, Uberaba, MG, Brazil), 200 µL of mineral oil and a gaseous mixture of 90% N_2_, 5% CO_2_ and 5% O_2_ and saturated humidity. The COC were transported to the laboratory in an oocyte transporter (TO-39 OLED, TED, Cravinhos, SP, Brazil). Oocytes remained in the same tubes, but they had their silicon lid switched on the moment they entered the incubator. *In vitro* maturation was 24 h in an incubator at 38.7ºC, in a gaseous atmosphere with 5% CO_2_ and saturated humidity.

### Evaluation of mitochondrial reorganization

After the IVM, in experiment 1 the oocytes (n= 390) were washed in TCM199 HEPES and incubated for 10 minutes in a solution of 1mg/mL of hyaluronidase in 400 µL of TCM199 HEPES at a temperature of 38.7ºC. Then, the cumulus cells were mechanically removed and washed in the same medium as before to have their IVM evaluated.

To valuation of mitochondrial reorganization, denuded oocytes were incubated in the dark for 30 minutes at 38.7ºC with Mitotracker Green FM (MT; Molecular Probes INC, USA) at a concentration of 250 nM in TCM199 HEPES and were evaluated by using a fluorescence microscope with an excitation wavelength of 490 nm and emission 516 nm. For the evaluation of cytoplasmic maturation, the method of mitochondrial reorganization was used. Oocytes with homogeneous dispersion and/or staining intensity associated with the central location of mitochondria had an indication of complete cytoplasmic maturation. Oocytes with mitochondria in the peripheral region of the ooplasm and/or with the presence of vacuoles had an indication of incomplete cytoplasmic maturation (Stojkovic et al., 2001).

### Evaluation of nuclear maturation

The same denuded oocytes (n=390) were co-incubated for 10 minutes, after 20 minutes in Mitotracker Green FM, in the dark with 10 µg/ml bisbenzimide (Hoechst 33342 – Sigma B5388) at 38.7°C and evaluated using a fluorescence microscope with an excitation wavelength of 365 nm and an emission wavelength of 410 nm. Oocytes were individually evaluated in 3µl drops of TCM199 HEPES. Those with extrusion of the first polar body were considered complete nuclear maturation.

### *In vitro* fertilization (IVF)

Semen straws of the same Braford bull with proven fertility were thawed in a water bath at 37°C for 30 seconds and homogenized. Subsequently, the semen was subjected to the washing selection method (Sanches et al., 2016). It was added 800 µL of TL Semen media (ABS Pecplan, Uberaba, MG, Brazil) plus semen. This mixture was subsequently centrifuged for five minutes at 500 x g. The pellet was resuspended in 800 µL of IVF media (ABS Pecplan, Uberaba, MG, Brazil) and recentrifuged again for five minutes at 500 x g. Then 120 μL pellet, resulting from the second centrifugation, was put in a 0.5 mL microtube and used 5 µL for each drop for IVF. The sperm cells and oocytes co-culture were performed for 24 h at 38.7 °C with saturated humidity and a gaseous atmosphere of 5% CO_2_.

### *In vitro* culture (IVC)

After 24 h of co-culture, the probable zygotes were denuded by pipetting and washed in 100 µL TL-semen wash media, then 50 µL TL-semen plus 50 µL SOF1 (ABS Pecplan, Uberaba, MG, Brazil), then 100 µL SOF1. Probable zygotes were transferred and allocated in 100μl drops of SOF1 medium under mineral oil and kept for a total of seven days in an incubator at 38,7ºC, 5% CO_2,_ 90% N_2_ and 5% O_2_. Uncleaved structures were discarded and 50% of the medium was replaced on day 3. Two days later (D5), 50% of the medium was replaced again with SOF 2 (ABS Pecplan, Uberaba, MG, Brazil).

### Cleavage and blastocyst rate

The number of cells at 72 hours post insemination (hpi) was evaluated. Structures with two or more cells were considered cleaved. Seven days after IVF, or 168 hpi, structures were evaluated regarding quantity and quality according to International Embryo Technology Society (IETS). Grades 1, 2, and 3 were counted for this variable. Only excellent quality embryos (grade 1) according to IETS were considered suitable for slow cryopreservation method (Sanches et al., 2016).

### Statistical analysis

Normality tests were performed with Shapiro-Wilk and Kolmogorov-Smirnov. On data considered parametric, it was performed ANOVA and the differences were compared by Tukey’s test. Variables that were considered nonparametric (medium and large follicles visualized on OPU, number of viable oocytes/donor/session and % of BCB – oocytes, limited to experiment one) went through Kruskal-Wallis test and a Chi-Square test was performed, and the differences were compared by Dunn’s test. Statistical Analysis System for Windows 9.0 (SAS 9.0, 2002) was used to verify statistical differences. The individual effect of the donor was included as a random effect. The effect of the farm was included as a covariate in the model. Means ± standard error of mean (SEM) was used. Significance was set at *P* ≤ 0.05 and tendencies were determined if P > 0.05 and ≤ 0.10.

## Results

**Figure 3:**
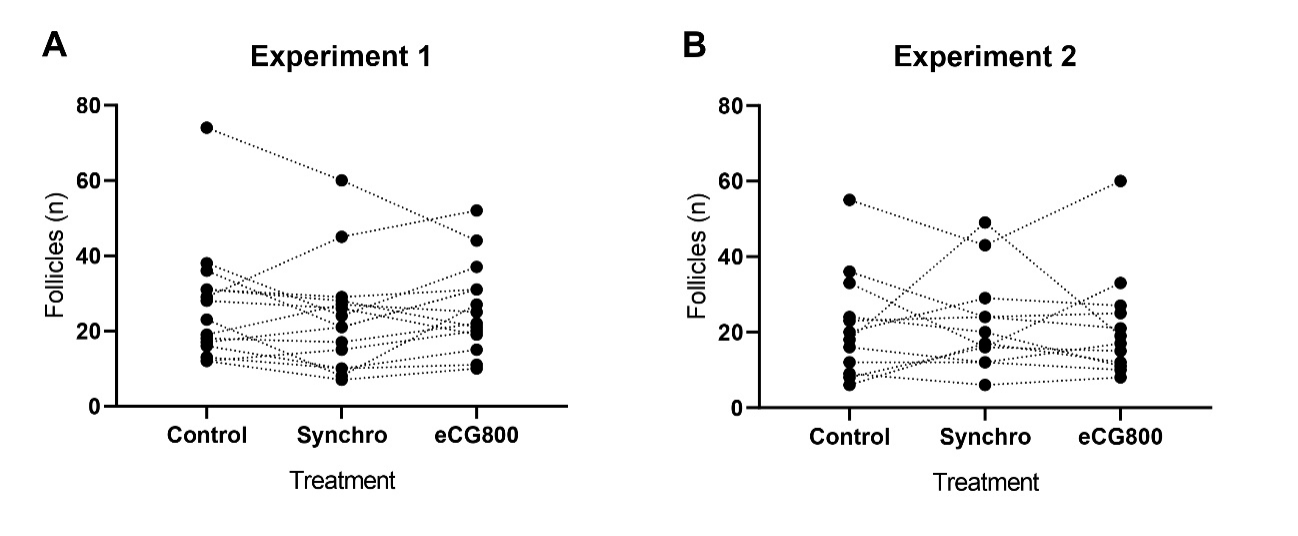
Distribution of follicle counts at the time of OPU in Braford cows subjected to Control, Synchro, or eCG800 treatments. Data includes all follicles visualized in both experiments at the time of aspiration.

The follicular diameter data from the accumulated experiments (n = 27) demonstrated that the number of all follicles visualized was similar among the treatments (*P* = 0.767). The medium (6 – 10 mm) and large (> 10mm) diameter follicles differed among the treatments (*P* < 0.05). For medium follicles, the eCG800 was significantly larger than the Control and Synchro groups. For large follicles, the eCG800 had a higher number of follicles compared to the Synchro group, without differing from the Control. Small (< 6mm) diameter follicles were similar to among groups (Table 1). The dispersion of the quantity of all follicles visualized at OPU in Braford cows subjected to different treatments (Control, Synchro and eCG800) in both experiments is shown in Figure 3.

**Table 1:** Proportion of small (< 6 mm) medium (6 – 10 mm) and large (> 10 mm) follicles visualized before ovum pick-up (OPU) in Braford cows subjected to different treatments (Control, Synchro and eCG800).

	**Control n (%)**	**Synchro n (%)**	**eCG800 n (%)**	***P-value***
No. OPU Sessions	27	27	27	
Small Follicles	623 ± 2.94 (94.82)	597 ± 2.60 (96.91)	516 ± 2.75 (78.90)	0.574
Medium Follicle	23b ± 0.20 (3.50)	11^b^ ± 0.13 (1.79)	92a ± 0.53 (14.07)	<0.0001
Large Follicles	11^ab^± 0.10 (1.68)	8^b^± 0.14 (1.30)	46^a^ ± 0.67 (7.03)	0.025
All Follicles	657 ± 2.88 (100)	616 ± 2.57 (100)	654 ± 2.48 (100)	0.987

a,b Different subscript letters demonstrate statistical difference. Data is shown as mean ± standard error (SEM).

The number of aspirated follicles/donor/session was similar to three treatments (*P* = 0.937). As well as the number of COC / donor / session (*P* = 0.973). Recovery (*P* = 0.394) and viable oocyte rates (*P* = 0.733) were similar (Table 2), as was the number of viable COCs/donor/session (P = 0.957).

**Table 2:** Results (mean ± SEM) of ovum pick-up (OPU), evaluation of oocyte competence and oocyte maturation in Braford cows subjected to different treatments (Control, Synchro and eCG800).

	**Control**	**Synchro**	**eCG800**	***P-value***
No. aspirated follicles / donor / session	22.22 ± 2.49	21.11 ± 2.38	22.04 ± 2.12	0.937
No. COC / donor / session	11.67 ± 2.36	11.63 ± 1.80	12.26 ± 2.19	0.973
Recovery rate (COC / aspirated follicles), %	46.19 ± 5.37	53.20 ± 5.61	49.96 ± 4.77	0.394
Viable oocyte rate, %	55.15 ± 6.52	60.86 ± 6.28	61.07 ± 5.14	0.733
No. viable COC / donor / session	8.19 ± 1.97	8.59 ± 1.70	8.96 ± 1.90	0.957
BCB + oocytes, %	75 ± 3.51	83.09 ± 10.9	87.9 ± 13.12	0.7244
Mitochondrial reorganization, %	59.68 ± 6.88	52.8 ± 7.02	49.26 ± 9.26	0.3424

COC: *Cumulus-oophorus* complexes; BCB: Brilliant cresyl blue. Data is shown as mean ± standard error (SEM).

In experiment 1, to oocyte competence using BCB staining, there was no difference found on BCB + (*P* = 0.724) or BCB – (*P* = 0.653; Table 2). To oocyte nuclear maturation, there were no differences for extrusion of the first polar body (*P* = 0.905) among the groups. For cytoplasmic maturation analyzed through mitochondrial reorganization, there was no difference among the groups (*P* = 0.342; Table 2).

**Figure 4:**
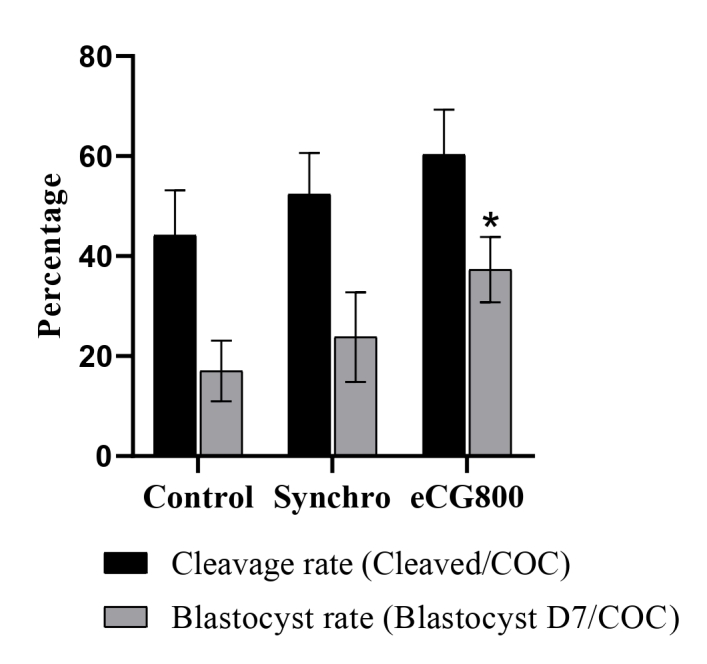
Cleavage and blastocyst rates in Braford cows are subjected to Control, Synchro, or eCG800 treatments. Cleavage rate (cleaved structures / total COC; P = 0.249). Blastocyst rate (blastocysts on day 7 / total COC) in Braford cows subjected to different treatments (Control, Synchro and eCG800). (*) Asterisk indicates statistical trend among groups (P=0.075).

The cleavage rate did not differ (P = 0.249) among Control (44.2%, n = 57/129), Synchro (52.4%, n = 66/126), and eCG800 (60.3%, n = 76/126) groups. Furthermore, the blastocyst rate on D7 showed tendency (P = 0.075) to improve from Control (17.05%, n = 22/129), to Synchro (23.81%, n = 30/126) to eCG800 (37.30%, n = 47/126; Figure 4).

Embryo grade 1 rate (*P* = 0.343) was similar among treatments, varying from 45.45% ± 7.93 (n = 10/22), 56.67% ± 9.42 (n = 17/30) to 55.31% ± 11.77 (n = 26/47) for Control, Synchro and eCG800 groups, respectively. There was a biological improvement in the number of blastocysts/donor/OPU session (P = 0.372) and freezable embryos/donor/OPU session (P = 0.301), with 1.83 ± 0.06 and 0.83 ± 0.03 for Control, 2.5 ± 0.06 and 1.42 ± 0.05 for Synchro, 3.91 ± 0.12 and 2.17 ± 0.07 for eCG800 group, respectively.

## Discussion

In this study, the administration of 800 IU of eCG before OPU in Braford cows increased the number of medium and large follicles compared to Control and Synchro groups. The total number of follicles, oocyte recovery, and viability rates were similar among treatments. There were no differences in oocyte competence parameters, including nuclear and cytoplasmic maturation and mitochondrial distribution. The eCG800 group showed a numerical tendency toward higher blastocyst rate. These results suggest that eCG promotes follicular growth without compromising oocyte quality.

Increased oocyte competence has been associated with medium follicles (Blondin et al., 2012; Ribas et al., 2018; Fernandes et al., 2021). The results of this study confirmed the positive effect of 800 IU eCG with more medium (6 to 10 mm) and large (> 10 mm) follicles at the time of OPU. Although eCG did not demonstrate changes in oocyte IVM, it tended to increase the blastocyst rate. This effect is particularly important in animals with a low number of available oocytes, as in the case of *Bos taurus taurus* donors, where superstimulation of follicular development may not increase the number of follicles, but rather improve oocyte quality and embryo production (Silva-Santos et al., 2014; Center et al., 2018; Ribas et al., 2018). The application of gonadotropic hormones induces follicular growth to obtain a greater number and proportion of follicles between 6 to 10 mm (Blondin et al., 2012). In this sense, donors treated with 800 IU of eCG increased the number of medium follicles at the time of OPU, similar data demonstrated by Vieira et al. (2014, 2016) using different doses of porcine FSH up in Holstein cattle. However, none of the treatments resulted in more aspirated follicles, more recovery rate, and viable oocytes.

The dispersion of germinal vesicle stages in follicles is heterogeneous (Lodde et al., 2007). The follicle diameter alone does not guarantee the stage of oocyte development. In different breeds on random days of the estrous cycle, the stages of the germinal vesicles were different (Soares et al., 2020a). However, wave synchronization and stimulation of follicular growth allows more competent oocytes at the same germinal vesicle stage (Soares et al., 2020b). Although oocytes derived from large follicles are expected to be more competent, in this study none of the treatments exceeded the results of the Control group. Furthermore, the development of oocyte competence rate (BCB+) in our study was higher (82.18%) than that found in other studies (Pujol et al., 2004; Alm et al., 2005; Silva et al., 2011; Otero et al., 2017). Despite that, nuclear and cytoplasmic maturation were not affected by any treatment when assessed through glucose-6-phosphate dehydrogenase enzymatic activity, first polar body extrusion, and mitochondrial reorganization.

It is established that mono ovulatory species, such as bovines, that the follicular environment impacts on oocyte development capacity. When performing OPU on random days of the estrous cycle, it is physiological to find a preovulatory follicle or one that becomes dominant soon after the deviation (i.e., 15 days of the luteal phase), which can be observed in the Control group, leading to more atretic events in the subordinate follicles. Once the dominant one is formed, it keeps growing and a final growth phase begins to allow endocrine signaling and ovulation (Sirard, 2011). It was demonstrated that 800 IU of eCG can improve fertilization rate and decrease the rate of polyspermy (Ribas et al., 2018) which can be numerically seen in our study by the upgrade on the cleavage rate, and furthermore, the blastocyst rate after seven days demonstrated an improvement with the eCG800 group showed a numerical tendency toward higher blastocyst rate. This may be due to a higher oocyte competence presented in larger than 6 mm follicle diameter. Considering that this effect can be attributed to the change in the profile of mRNA of genes related to the quality of oocytes (Fernandes et al., 2021), the use of 800 IU of eCG or follicular wave synchronization can change patterns of COCs molecular phenotype. However, new studies with a larger number of animals should be carried out to demonstrate the effect of the 800 IU dose of eCG on the production and quality of bovine embryos.

Oocyte recovery rate was not affected by follicular diameter in the present study, although it has been reported in other studies (Vieira et al., 2014; Ribas et al., 2018) that the increased volume and viscosity of follicular fluid and the greater intra-follicular pressure of large follicles submitted to prior stimulation may hamper COC recovery. The number of fresh transferable blastocysts/donor/OPU more than doubled when donors were stimulated with 800 IU of eCG and increased 36.6% when donors had their follicular wave growth synchronized. Studies suggest that adjustments can be done to improve OPU programs, including cycle synchronization and hormonal stimulation to achieve better blastocyst yield (Cavalieri et al., 2018; Seneda et al., 2020). In addition, it is important to emphasize that in this study, the crossover design ensured that all animals were subjected to all treatments, minimizing the influence of individual variables, which strengthens the results obtained. Considering only grade 1 embryos can be submitted to slow freezing method (Sanches et al., 2016; direct transfer), there also was an improvement of 71% from Control to wave synchronization and a little more than 2.5 times from Control to donors treated with 800 IU of eCG which shows the advantages of synchronization and stimulation with 800IU of eCG prior to OPU.

## Conclusion

Follicular wave synchronization with 800 IU eCG 72 hours before OPU increased the number of medium and large follicles in Braford cows without affecting oocyte recovery, viability, or developmental competence. Furthermore, the 800 IU eCG tended to have a higher blastocyst rate compared to the Control group, as well as grade 1 embryos. Therefore, this protocol may be a useful tool to optimize OPU/IVP results in Braford cows.

## Data Availability

Research data is only available upon request.
